# Embodiment Is Related to Better Performance on a Brain–Computer Interface in Immersive Virtual Reality: A Pilot Study

**DOI:** 10.3390/s20041204

**Published:** 2020-02-22

**Authors:** Julia M. Juliano, Ryan P. Spicer, Athanasios Vourvopoulos, Stephanie Lefebvre, Kay Jann, Tyler Ard, Emiliano Santarnecchi, David M. Krum, Sook-Lei Liew

**Affiliations:** 1 Neural Plasticity and Neurorehabilitation Laboratory, Neuroscience Graduate Program, University of Southern California, Los Angeles, CA 90089, USA; juliaang@usc.edu; 2 Institute for Creative Technologies, University of Southern California, Playa Vista, CA 90094, USA; ryanspicer@gmail.com (R.P.S.); krum@ict.usc.edu (D.M.K.); 3 Neural Plasticity and Neurorehabilitation Laboratory, Division of Occupational Science and Occupational Therapy, University of Southern California, Los Angeles, CA 90089, USA; thanos@vourvopoulos.com (A.V.); slefebvr@usc.edu (S.L.); 4 USC Stevens Neuroimaging and Informatics Institute, Department of Neurology, University of Southern California, Los Angeles, CA 90033, USA; kay.jann@loni.usc.edu (K.J.); tyler.ard@loni.usc.edu (T.A.); 5 Berenson-Allen Center for Non-Invasive Brain Stimulation and Division of Cognitive Neurology, Beth Israel Deaconess Medical Center, Harvard Medical School, Boston, MA 02215, USA; esantarn@bidmc.harvard.edu

**Keywords:** brain–computer interface, neurofeedback, immersive virtual reality, head-mounted display, electroencephalography, presence, embodiment

## Abstract

Electroencephalography (EEG)-based brain–computer interfaces (BCIs) for motor rehabilitation aim to “close the loop” between attempted motor commands and sensory feedback by providing supplemental information when individuals successfully achieve specific brain patterns. Existing EEG-based BCIs use various displays to provide feedback, ranging from displays considered more immersive (e.g., head-mounted display virtual reality (HMD-VR)) to displays considered less immersive (e.g., computer screens). However, it is not clear whether more immersive displays improve neurofeedback performance and whether there are individual performance differences in HMD-VR versus screen-based neurofeedback. In this pilot study, we compared neurofeedback performance in HMD-VR versus a computer screen in 12 healthy individuals and examined whether individual differences on two measures (i.e., presence, embodiment) were related to neurofeedback performance in either environment. We found that, while participants’ performance on the BCI was similar between display conditions, the participants’ reported levels of embodiment were significantly different. Specifically, participants experienced higher levels of embodiment in HMD-VR compared to a computer screen. We further found that reported levels of embodiment positively correlated with neurofeedback performance only in HMD-VR. Overall, these preliminary results suggest that embodiment may relate to better performance on EEG-based BCIs and that HMD-VR may increase embodiment compared to computer screens.

## 1. Introduction

Neurofeedback training produces beneficial changes in motor function and has been shown to be successful in motor rehabilitation for clinical populations, such as individuals with stroke [1]. Electroencephalography (EEG) can be used to measure brain activity used by brain–computer interfaces (BCI) to provide sensory feedback to reward specific brain activity patterns. This feedback can then be used to control a robotic or computerized device (e.g., movement of an object on a computer screen) to train individuals to control their own brain activity. BCIs designed for the rehabilitation of individuals with severe motor impairment attempt to “close the loop” between motor commands and sensory feedback by providing supplemental sensory information when individuals successfully establish specific brain patterns. Successfully closing the loop is achieved when individuals perceive the control of supplemental sensory information as their own (i.e., embodiment). Perceived embodiment can influence an individual’s sense of agency [2] where greater embodiment may result in a sense of increased control, but a lack of embodiment may result in a sense of decreased control or distress and lead to a distortion of capabilities [3].

Given that individuals with severe motor impairment cannot generate active volitional movement, a primary neurofeedback approach is to use imagined movement (i.e., motor imagery) to drive the BCI. Motor imagery (MI) is thought to engage areas that modulate movement execution [4,5,6]. MI has been shown to be an effective intervention for motor rehabilitation, especially when it is coupled with physical practice [7,8]. Previous work has shown that BCIs employing MI can produce clinically meaningful improvements in motor function in individuals with motor impairments [9,10,11,12,13,14]. These BCIs have used a variety of displays to provide feedback, ranging from devices that provide an immersive and compelling experience (e.g., projected limbs, robotic orthoses, or exoskeletons) [1,10,11,12] to devices that are considered less immersive (e.g., computer screens) [9,13]. Recently, BCIs have also begun to incorporate immersive virtual reality using a head-mounted display (HMD-VR) in order to provide a more immersive and realistic environment [15] and to provide more biologically relevant feedback [16]. However, it is not known whether HMD-VR improves neurofeedback performance compared to feedback provided on a screen. It is also unclear whether neurofeedback provided in HMD-VR increases embodiment, which in the context of HMD-VR can be defined as the perceptual ownership of a virtual body in a virtual space [17,18], compared to screen-based neurofeedback. 

Studies have shown that HMD-VR facilitates the embodiment of a virtual body and that the observation of this virtual body in the first person perspective is enough to induce a strong feeling of embodiment for the virtual body’s actions [17,19,20,21,22]. In HMD-VR, individuals exhibit behaviors that match those of a digital self-representation, such as overestimating object sizes when an adult has been given a virtual child body [17] or exhibiting a reduction in implicit racial bias when given a body of a different race [23]. Initially coined the Proteus effect [19], this sense of embodiment that arises from viewing a virtual limb has the potential to alter one’s own neurophysiology and behavior. Regarding motor behavior, an increased level of embodiment has been shown to be related to increased sensorimotor rhythms (SMR) desynchronization [24]. In related work, observing the actions of virtual limbs in virtual reality has been shown to increase SMR desynchronization [25]. In addition, the immersive nature of HMD-VR has also been shown to increase an individual’s sense of presence in the virtual environment [24], which in the context of HMD-VR can be defined as the illusion of actually being present in the virtual environment [26]. It is also unclear whether neurofeedback provided in HMD-VR increases one’s feeling of presence compared to screen-based neurofeedback. Here we examined the role of both embodiment and presence on neurofeedback performance using HMD-VR versus a computer screen through the use of qualitative questionnaires previously used to measure these concepts [17,27,28]. 

We have created a hybrid brain–computer interface for individuals with severe motor impairments called REINVENT (Rehabilitation Environment using the Integration of Neuromuscular-based Virtual Enhancements for Neural Training), which provides brain (EEG) and/or muscle (electromyography (EMG)) neurofeedback in HMD-VR. Although we designed REINVENT as an EEG-based BCI device for individuals with severe motor impairments, in this pilot study, we first wanted to examine whether providing neurofeedback in HMD-VR improves neurofeedback performance compared to receiving the same neurofeedback on a computer screen in healthy adults. Furthermore, we wanted to examine whether there were differences in the levels of embodiment and presence induced by HMD-VR versus a computer screen and how individual differences in these features relate to neurofeedback performance in each environment. As embodiment and presence play an important role in increasing SMR desynchronization and HMD-VR induces high levels of embodiment and presence, we predicted that participants would show better neurofeedback performance in an HMD-VR environment compared to a computer screen, and that improved performance would be related to increased embodiment and presence.

## 2. Materials and Methods

### 2.1. Participants

Twelve healthy participants were recruited for this experiment (7 females/5 males; age: M = 24.4 years, SD = 2.7 years) where all participants underwent the same experimental design (see Section 2.3). Eligibility criteria included healthy, right handed individuals, and informed consent was obtained from all participants. Eight participants reported being naive to immersive virtual reality using a head-mounted display; the four participants with previous use of head-mounted displays reported using the device no more than four times. The experimental protocol was approved by the University of Southern California Health Sciences Campus Institutional Review Board and performed in accordance with the 1964 Declaration of Helsinki.

### 2.2. REINVENT Hardware, Software, Online Processing, and Data Integration

**Figure 1:**
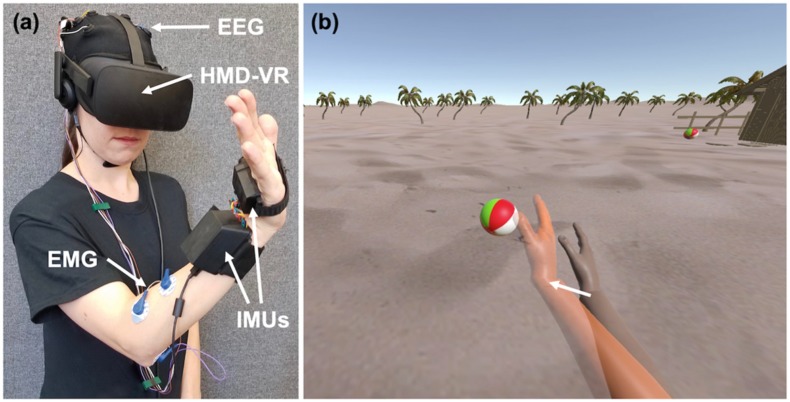
Example of the REINVENT system. (**a**) REINVENT hardware used here is composed of electroencephalography (EEG), electromyography (EMG), inertial measurement units (IMUs), and a head-mounted display virtual reality (HMD-VR) system. Written informed consent for the publication of this image was obtained from the individual depicted. (**b**) The environment participants observed on both a computer screen and in HMD-VR; arm movements are goal-oriented such that when the arm reaches a target position, it interacts with an object (e.g., hitting a beach ball). On EEG blocks (Screen, HMD-VR), participants would attempt to move their virtual arm (right arm) to the orange target arm (left arm) by thinking about movement. On the IMU block, the virtual arm would match participants actual arm movements.

The REINVENT system (see Figure 1a for an example) is a brain–computer interface (BCI) composed of four main components: EEG, EMG, an inertial measurement unit (IMU), and a HMD-VR system [29]. While the current study only utilized the EEG and IMU components, we describe the entire system here.

#### 2.2.1. Electroencephalography (EEG) and Electromyography (EMG)

The EEG/EMG component of REINVENT is composed of hardware from OpenBCI (www.openbci.com), a low-cost solution for measuring brain and muscle activity. The EEG component consists of reusable dry EEG electrodes, and the EMG component consists of snap electrode cables connected to mini disposable gel electrodes (Davis Medical Electronics, Inc.). Both EEG and EMG wires were connected to a 16-channel, 32-bit v3 processor (Cyton + Daisy Biosensing Open BCI Board) and sampled at 125 Hz. Twelve EEG locations based on the international 10-20 system [30] and concentrated over the prefrontal and motor cortex are used to record brain activity (F3, F4, C1, C2, C3, C4, CP1, CP2, CP5, CP6, P3, and P4); however, in the current study we were primarily interested in channels closest to the left motor network (i.e., C1, C3, and CP1). Ground and reference electrodes are located at the right and left earlobes, respectively. EMG is recorded from four electrodes placed on the wrist flexors and extensors on the muscle bellies of the right forearm, with a reference electrode on the bony prominence of the elbow. In the current experiment, muscle activity from EMG was collected during the experiment to examine EEG–EMG coherence, but as these analyses are beyond the scope of the current study, they are not included here.

Custom software is used to control the BCI and provide users with real-time neurofeedback of a virtual arm. The neurofeedback, composed of the sum desynchronization from C1, C3, and CP1, was used to drive the movement of a virtual right arm towards a target arm. EEG signals were recorded from electrodes of interest over the left motor cortex (i.e., C1, C3, and CP1, based on the international 10-20 system) with both ear lobes used as the ground and reference electrodes, and sent to the REINVENT software. Data processing occurred online. Individual channels were high-pass filtered using a second order Butterworth filter with a cutoff of 3 Hz, and a sliding window consisting of 125 incoming samples were fast Fourier transformed (FFT). Power was then computed between the frequency ranges of 8-24 Hz, capturing the broad activity in alpha and beta bands that may correspond to motor imagery (i.e., sensorimotor desynchronization). The virtual arm direction updated every second and moved towards the target in response to sensorimotor desynchronization, measured as a decrease in amplitude compared to the baseline recording of the left sensorimotor area (i.e., the combined C1, C3, and CP1).

#### 2.2.2. Inertial Measurement Unit (IMU)

The IMU component of REINVENT is composed of two nine degrees of freedom IMUs, with one placed on the hand and the other placed on the wrist of the right arm. To foster a sense of embodiment between the participant and the virtual arm, the REINVENT system allows for the participant’s own arm movements to be recorded. Before beginning this experiment, the participant’s arm was passively moved by the experimenter, and the virtual representation of the arm was shown on the computer screen and in HMD-VR. In this way, a sensorimotor contingency was developed between the participant’s own arm and the virtual arm they were subsequently asked to control.

### 2.3. Experimental Design

**Figure 2:**
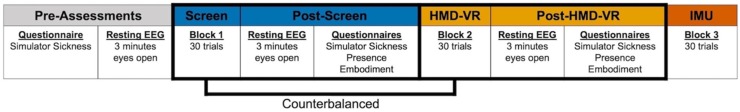
Experimental timeline. Prior to the experimental blocks, participants completed a questionnaire relating to simulator sickness and then completed a resting EEG recording for three minutes with eyes open. Participants then completed the three experimental blocks where the first two blocks were counterbalanced; during Blocks 1 and 2 (Screen, HMD-VR), participants were asked to think about movement in order to move their virtual arm to a virtual target arm on either a computer screen or in HMD-VR. After the Screen condition and after the HMD-VR condition, participants completed a resting EEG recording for three minutes with eyes open and then completed a series of questionnaires relating to simulator sickness, presence, and embodiment. During Block 3 (IMU), participants were asked to move their physical arm to a virtual target arm in HMD-VR, as a control condition.

We used a within-subject experimental design where all participants underwent the same protocol (Figure 2). Prior to the experiment, participants underwent pre-assessments that included a simulator sickness questionnaire (see Section 2.6) and a resting EEG baseline recording. The resting EEG baseline recording lasted three minutes and was recorded while the HMD-VR was removed. For the duration of the recording, participants were instructed to keep their eyes open and fixed on a location at the center of the computer screen and asked to think about a stationary object and to stay as still as possible. The recording was used to provide the baseline EEG values for the experiment. Following the resting EEG baseline recoding, participants completed three blocks of 30 trials (90 trials in total) where each block was a separate condition. The conditions were (1) controlling the virtual arm with brain activity on the computer screen (Screen), (2) controlling the virtual arm with brain activity in a head-mounted display virtual reality (HMD-VR) system, and (3) controlling the virtual arm with actual arm movements in a head-mounted display (IMU). Participants completed the conditions in the following block order: Block 1 (Screen), Block 2 (HMD-VR), Block 3 (IMU), with Blocks 1 and 2 (Screen, HMD-VR) counterbalanced. In this experiment, the IMU condition strictly provided a control condition of real movement instead of neurofeedback; these data are briefly reported but not focused on in this paper. 

Before starting the experimental conditions, participants were given instructions on how to control their virtual arm (i.e., “You will see two right arms. One is orange and that is the target arm that moves to different positions. The other is your arm. We want you to move it to match the target arm’s position. You can move your arm in two ways. First, you will complete 60 trials of moving the virtual arm with just your thoughts by thinking about moving; 30 of the trials will be on the computer screen, without the head-mounted display, and 30 trials will be with the head-mounted display. Then you will complete 30 trials of moving the virtual arm using your actual arm movements.”). Instructions were repeated at the start of each condition. For each EEG neurofeedback condition (Screen, HMD-VR), participants were instructed to stay as still as possible. After the completion of each EEG neurofeedback condition, a resting-EEG acquisition of three minutes was recorded while the HMD-VR was removed; participants were again instructed to keep their eyes open and fixed on the center of the screen for the duration of the recording. For the duration of the experiment, participants were seated and asked to rest their hands comfortably on a pillow placed across their lap.

### 2.4. Individual Trials

At the start of each trial, a target arm animated a wrist extension pose in one of three target positions. Once the target arm stopped moving, participants were instructed to move their virtual arm to match the position of the target arm given the current condition (i.e., in the case of the two EEG neurofeedback conditions (Screen, HMD-VR), and they were asked to think about moving; in the case of the IMU condition, they were asked to actually move their arm to the target location). During the EEG neurofeedback condition trials, the virtual hand incremented either forward or backward, as determined by the sum of the three channel EEG desynchronization compared to baseline. Most of the time, the EEG activity was significantly above or below the baseline; however, if the sensorimotor activity was hovering around the baseline, the arm would move back and forth. The duration of each trial was 15 seconds. If the target arm was reached within this time constraint, a successful auditory tone was played; however, if the target arm was not reached, then an unsuccessful auditory tone was played. At the completion of each trial, the target and virtual arms returned to their starting position.

### 2.5. Displays and Neurofeedback

For all conditions, participants observed the virtual arm from a first person perspective. For the HMD-VR and IMU conditions, we used the Oculus CV1, which includes positional and rotational tracking to display the stimuli. For the Screen condition, we used a 24.1 inch, 1920 × 1200 pixel resolution computer monitor (Hewlett-Packard) to display the stimuli. In both displays, participants observed a scene that included two virtual arms: (1) one virtual arm that represented the participant’s own arm and (2) a second virtual arm, colored in orange, that provided different target arm positions that participants were asked to move their own virtual arm towards (Figure 1b). For all conditions, the target arm generated wrist extension movements to different target locations; participants were either asked to either think about moving their arm to these locations (EEG neurofeedback conditions: Screen, HMD-VR) or actually move their arm to these locations (IMU condition). In the IMU condition of the experiment, participants were required to actually perform wrist extension movements to match the virtual arm in the HMD-VR display.

### 2.6. Subjective Questionnaires

Prior to the experiment, participants were given a series of standard questions about their baseline comfort levels (simulator sickness questionnaire; adapted from Kennedy et al. (1993)) [31]. After participants completed each EEG neurofeedback condition (Screen, HMD-VR), they were given the same simulator sickness questionnaire to examine changes following each block. Responses were reported on a 0 to 3-point scale, and questions were collapsed along three main features: Nausea, Oculomotor, and Disorientation. In addition, after completing both the Screen and HMD-VR conditions, participants were also asked questions pertaining to their overall sense of presence and embodiment in each respective environment. The Presence Questionnaire was adapted from Witmer and Singer (1998) [27] and revised by the UQO Cyberpsychology Lab (2004) and asked participants a series of questions to gauge their sense of presence in each environment. Responses were reported on a 1 to 7-point scale, and questions were collapsed along five main features: Realism, Possibility to Act, Quality of Interface, Possibility to Examine, and Self-Evaluation of Performance. The Presence Questionnaire is a validated questionnaire [32] that has been used in research using HMD-VR [33]. The Embodiment Questionnaire was adapted from Bailey et al. (2016) [28] and Banakou et al. (2013) [17] and asked participants a series of questions to gauge their sense of embodiment. Responses were reported on a 1 to 10-point scale and questions were averaged to generate an overall Embodiment feature. In addition, we also collapsed questions relating to either Self Embodiment or Spatial Embodiment to generate two embodiment sub-features. Self Embodiment describes the extent to which participants felt the virtual arm was an extension of their own arm, and Spatial Embodiment describes the extent to which participants felt that they were in the virtual environment. Table 1 includes individual questions asked on the Embodiment Questionnaire.

### 2.7. Analyses

#### 2.7.1. Post-Hoc EEG Analysis on Activity During Task

In addition to the online processing (see Section 2.2), post-hoc EEG signals were processed offline using MATLAB® (R2017a, The MathWorks, MA, USA) with the EEGLAB toolbox [34]. After importing the data and channel information, a high-pass filter at 1 Hz was applied to remove the baseline drift followed by line-noise and harmonics removal at 60 Hz. Furthermore, bad channels were rejected, while any potential missing channels were interpolated before the re-referencing stage. Additionally, all channels were re-referenced to the average. Next, data epoching was performed by extracting the trials from the EEG neurofeedback conditions (Screen, HMD-VR) for each participant. Artifact rejection was performed using independent component analysis (ICA) over the epoched data and visual inspection of the time-series. Finally, the baseline data (180 seconds) were extracted from the resting-state session that occurred before the task.

For computing the average spectral power, Welch’s method for power spectral density (PSD) of the power spectrum [35] was used across the online frequency range (8–24 Hz) and for the alpha (8–12 Hz) and beta (13–24 Hz) bands. PSD was extracted from both the epoched motor-related data and the baseline. As there was only one movement detection with one degree of freedom, classifier training did not take place. We used the target location C3 to represent the primary motor cortex; thus, the band power was extracted over the C3 electrode location and calculated using the following formula:

(1) $$PSD_{Band}=PowerC3_{Motor\;Activity}-\;PowerC3_{Baseline},$$

Similarly, for analyzing the primary ipsilateral motor cortex, we used the target location C4 [36,37,38].

#### 2.7.2. Statistical Analysis

All data used in statistical results can be found in the supplementary materials (supporting datasets). Statistical analysis for neurofeedback performance, subjective experience from questionnaires, and EEG activity during the task was analyzed using the statistical package R (Version 3.2.2) using R Studio (Version 1.1.423). For each variable, we checked for normality using a Shapiro–Wilk test. To assess statistical differences in performance, subjective experience, and average spectral power during the task between the two EEG conditions (Screen, HMD-VR), a paired t-test was performed on each measure found to be normally distributed, and a Mann–Whitney U test was performed on each measure found to not be normally distributed. Means (M), standard deviations (SD), and skewness are reported for each measure (Supplementary Table S1). To confirm that neurofeedback based on motor imagery was successfully used to increase performance, we ran a simple linear regression on neurofeedback performance based on PSD. Lastly, we examined the relationship between neurofeedback performance and responses from the Presence Questionnaire and the Embodiment Questionnaire using regression analysis. For both questionnaires, we first tested for normality using a Shapiro–Wilk normality test. For the Presence Questionnaire, we ran a multiple regression analysis on neurofeedback performance based on the five presence features for each condition (Screen, HMD-VR). For the Embodiment Questionnaire, we first ran a simple linear regression analysis on neurofeedback performance based on the overall Embodiment feature for each condition. Then, we ran a multiple regression analysis on neurofeedback performance based on the two embodiment sub-features (Self Embodiment and Spatial Embodiment) for each condition. For all regression analyses, adjusted R^2^ is reported. Finally, all participants completed the control IMU condition with 100% accuracy, and therefore this condition is not included in further analysis.

## 3. Results

### 3.1. Relationship Between Power Spectral Density and Neurofeedback Performance

**Figure 3:**
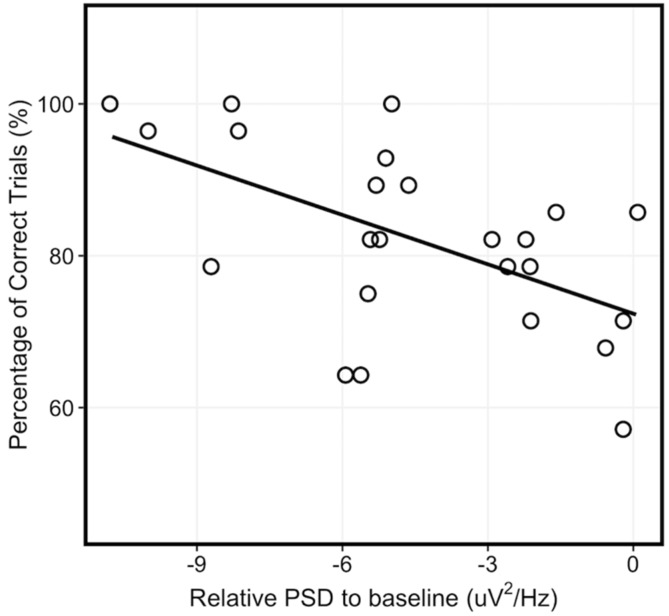
Relationship between power spectral density and neurofeedback performance. There was a significant relationship between power spectral density (PSD) and neurofeedback performance across the EEG neurofeedback conditions (combined Screen and HMD-VR).

To confirm the relationship between PSD in the 8-24 Hz frequency range and the corresponding neurofeedback performance, we ran a simple linear regression of neurofeedback performance based on PSD across the combined EEG neurofeedback conditions (i.e., Screen and HMD-VR). We found a significant relationship between PSD and neurofeedback performance (Figure 3; F_(1,22)_ = 9.328, p = 0.006; R^2^ = 0.266) where an increased sensorimotor desynchronization corresponded to better neurofeedback performance.

### 3.2. Comparison of Neurofeedback Performance and Time to Complete Successful Trials Between Screen and HMD-VR

**Figure 4:**
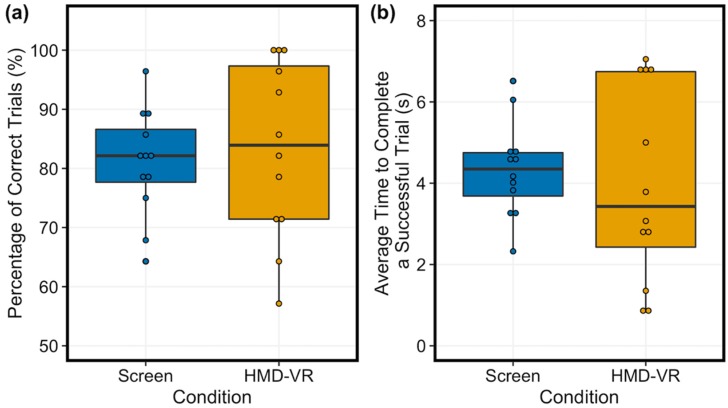
Average performance on trials and time to complete successful trials between conditions. (**a**) The analysis showed no significant differences in performance between Screen (left, blue) and HMD-VR (right, yellow) conditions. (**b**) The analysis showed no significant differences in time on successful trials between Screen (left, blue) and HMD-VR (right, yellow) conditions.

The proportion of correct trials completed was similar between the two conditions (Figure 4a; t(11) = −0.46, p = 0.656, d = 0.19; Screen: M = 80.95%, SD = 9.1%, and HMD-VR: M = 83.33%, SD = 14.9%). These results suggest that participants seemed to perform similarly independent of whether neurofeedback was provided in HMD-VR or on a computer screen. In addition, the time to complete each of the successful trials was also similar between the two conditions (Figure 4b; t(11) = 0.54, p = 0.597, d = 0.19; Screen: M = 4.347 s, SD = 1.17 s, and HMD-VR: M = 3.996 s, SD = 2.41 s). These results suggest that when participants were able to increment the virtual arm towards the target with their brain activity, the efficiency of control was similar whether viewing the arm in the HMD-VR environment or on a computer screen. The distribution of the data was not significantly different from a normal distribution for neurofeedback performance and time to complete successful trials.

### 3.3. Comparison of Power Spectral Density Between Screen and HMD-VR

**Figure 5:**
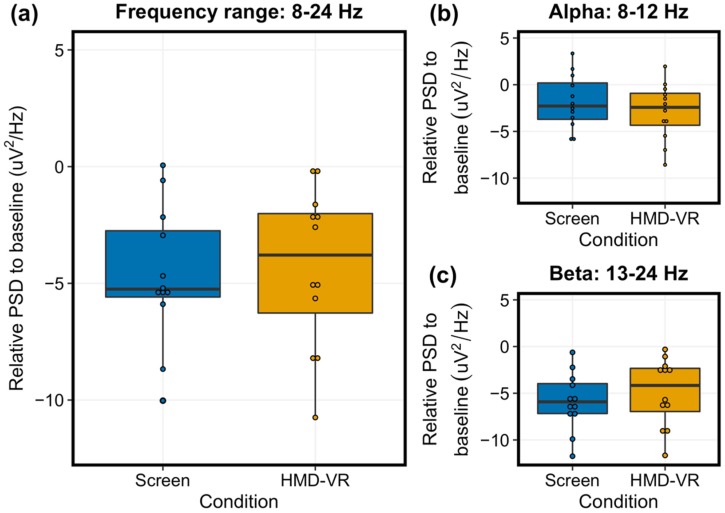
Average power spectral density during trials between conditions. (**a**) The relative group-level PSD for the target electrode C3, representing the left motor cortex (8-24 Hz) between the Screen (left, blue) and HMD-VR (right, yellow) conditions was not significantly different. (**b**) The relative group-level alpha between the Screen (left, blue) and HMD-VR (right, yellow) conditions was also not significantly different. (**c**) The relative group-level beta between the Screen (left, blue) and HMD-VR (right, yellow) conditions was also not significantly different.

Similar to the neurofeedback performance results, we did not find significant differences in group-level PSD between the Screen and HMD-VR conditions across the 8–24 Hz frequency range (Figure 5a; t(11) = 0.475, p = 0.644, d = 0.12; Screen: M = −4.69, SD = 2.96, and HMD-VR: M = −4.32, SD = 3.41). We also explored alpha and beta bands separately and did not find significant differences in group-level PSD between the Screen and HMD-VR conditions in either band (alpha: Figure 5b, t(11) = 1.363, p = 0.200, d = 0.35, Screen: M = −1.84, SD = 2.90, and HMD-VR: M = −2.89, SD = 3.04; beta: Figure 5c, t(11) = −1.141, p = 0.278, d = 0.29, Screen: M = −5.88, SD = 3.08, and HMD-VR: M = −4.92, SD = 3.63). This further suggests that participants had similar levels of sensorimotor activity whether neurofeedback was provided in HMD-VR or on a computer screen. The distribution of the data was not significantly different from a normal distribution for each power spectral density variable. Additionally, we have included two supplementary figures reporting individual participant EEG activity in alpha and beta bands for both C3 (Supplementary Figure S1; contralateral to and controlling of the virtual hand) and C4 (Supplementary Figure S2; ipsilateral to the virtual hand) recordings.

### 3.4. Comparison of Simulator Sickness Between Screen and HMD-VR

We checked for normality for each feature obtained by the simulator sickness questionnaire (i.e., Nausea, Oculomotor, Disorientation). For two of the features, the distribution of the data was significantly different from a normal distribution; thus, we used a nonparametic Mann–Whitney U test for each of the comparisons.

We found no significant differences in reports of simulator sickness between the Screen (Nausea: M = 1.59, SD = 8.94; Oculomotor: M = 9.48, SD = 12.15; Disorientation: M = 4.64, SD = 17.13) and the HMD-VR (Nausea: M = 2.39, SD = 5.93; Oculomotor: M = 9.45, SD = 9.76; Disorientation: M = 3.48, SD = 8.65) conditions (Nausea: U = 66, p = 0.730, d = 0.10; Oculomotor: U = 68, p = 0.837, d = 0.00; Disorientation: U = 67.5, p = 0.745, d = 0.09). These results suggest that HMD-VR neurofeedback does not cause additional adverse effects beyond using a computer screen in healthy individuals.

### 3.5. Comparison of Presence and Embodiment Between Screen and HMD-VR

We checked for normality for each feature obtained by the Presence Questionnaire (i.e., Realism, Possibility to Act, Quality of Interface, Possibility to Examine, and Self-Evaluation of Performance) and Embodiment Questionnaire (i.e., Embodiment, Self Embodiment, Spatial Embodiment). For each feature, the distribution of the data was not significantly different from a normal distribution.

There was a significant difference in reports of embodiment between the two conditions (t(11) = −2.21, p = 0.049, d = 0.48; Screen: M = 4.68, SD = 1.27, and HMD-VR: M = 5.4, SD = 1.71) where individuals reported higher levels of Embodiment in the HMD-VR condition. We then examined the sub-features of embodiment and found a significant difference in reports of Spatial Embodiment between the two conditions (t(11) = −3.77, p = 0.003, d = 0.87; Screen: M = 3.60, SD = 2.04, and HMD-VR: M = 5.35, SD = 2.00) where individuals reported higher levels of Spatial Embodiment in the HMD-VR condition. However, there was no significant difference in reports of Self Embodiment between the two conditions (t(11) = −0.10, p = 0.922, d = 0.03; Screen: M = 5.39, SD = 1.17, HMD-VR: M = 5.43, SD = 1.76). These results suggest that neurofeedback presented in a first person perspective in HMD-VR may increase one’s feeling of embodiment compared to neurofeedback presented on a computer screen.

In addition, there were no significant differences between reports of presence in the two conditions (Realism: t(11) = −1.95, p = 0.078, d = 0.47, Screen: M = 30.00, SD = 6.35, HMD-VR: M = 33.00, SD = 6.40; Possibility to Act: t(11) = −1.37, p = 0.199, d = 0.44, Screen: M = 18.17, SD = 3.70, HMD-VR: M = 19.92, SD = 4.19; Quality of Interface: t(11) = − 0.62, p = 0.548, d = 0.19, Screen: M = 12.83, SD = 3.07, HMD-VR: M = 13.42, SD = 2.97; Possibility to Examine: t(11) = − 2.01, p = 0.070, d = 0.72, Screen: M = 13.17, SD = 2.59, HMD-VR: M = 14.92, SD = 2.27; Self-Evaluation of Performance: t(11) = −1.24, p = 0.241, d = 0.49, Screen: M = 10.0, SD = 1.95, HMD-VR: M = 11.00, SD = 2.13). This suggests that HMD-VR neurofeedback may specifically increase embodiment but not presence in healthy individuals.

### 3.6. Relationship Between Embodiment, Presence, and Neurofeedback Performance

**Figure 6:**
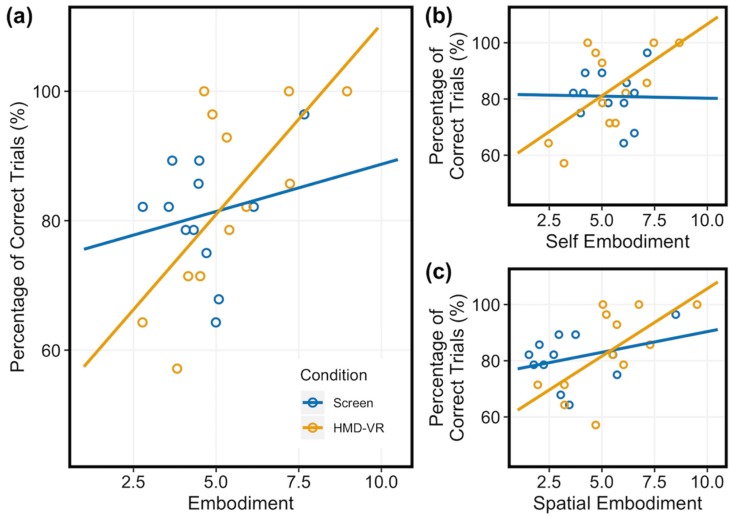
Relationship between subjective experience and neurofeedback performance in Screen (blue) and HMD-VR (yellow). Participants reported their level of Embodiment on a scale from 1 to 10 (Table 1). (**a**) Embodiment: For the HMD-VR condition, embodiment was significantly related to performance. However, for the Screen condition, embodiment did not significantly relate to neurofeedback performance. (**b**) Self Embodiment and (**c**) Spatial Embodiment: For the HMD-VR condition, we found a near significant relationship between the two embodiment sub-features and neurofeedback performance. However, for the Screen condition, we did not find a significant relationship between the two embodiment sub-features and neurofeedback performance.

We next examined whether individual differences in embodiment related to neurofeedback performance for each condition. We ran a simple linear regression of neurofeedback performance based on the overall Embodiment feature. For the HMD-VR condition, we found a significant relationship between embodiment and neurofeedback performance (F_(1,10)_ = 8.293, p = 0.016; R^2^ = 0.399). However, for the Screen condition, we did not find a significant relationship between embodiment and neurofeedback performance (F_(1,10)_ = 0.434, p = 0.525; R^2^ = −0.054). These results suggest that level of embodiment is specifically related to neurofeedback performance only in HMD-VR and not on a computer screen (Figure 6a). 

To better understand whether specific sub-features of embodiment also related to neurofeedback performance, we then examined if participants’ levels of self and spatial embodiment related to their neurofeedback performance for each condition (Screen, HMD-VR). We ran a multiple linear regression of neurofeedback performance based on the two embodiment sub-features (i.e., Self Embodiment, Spatial Embodiment). For the HMD-VR condition, we found a near significant relationship between the two embodiment sub-features and neurofeedback performance (F_(2,9)_ = 3.858, p = 0.0617; R^2^ = 0.342). For the Screen condition, we did not find a significant relationship between the two embodiment sub-features and neurofeedback performance (F_(2,9)_ = 0.706, p = 0.519; R^2^ = −0.056). These results further suggest that level of embodiment is specifically related to HMD-VR neurofeedback performance. Figure 6b,c shows regression lines for both Self Embodiment and Spatial Embodiment, respectively. 

Although there were no differences in presence between the Screen and HMD-VR conditions, we also explored whether individual differences in presence related to neurofeedback performance for each condition (Screen, HMD-VR). We ran a multiple linear regression of neurofeedback performance based on the five presence features (i.e., Realism, Possibility to Act, Quality of Interface, Possibility to Examine, and Self-Evaluation of Performance). We did not find a significant relationship between the five presence features and neurofeedback performance for either the Screen or HMD-VR condition (HMD-VR: F_(5,6)_ = 0.476, p = 0.452; R^2^ = 0.039; Screen: F_(5,6)_ = 0.840, p = 0.567; R^2^ = −0.078). These results suggest that the level of presence does not seem to be significantly related to either HMD-VR or computer screen neurofeedback performance.

## 4. Discussion

The current pilot study examined whether neurofeedback from a motor-related brain–computer interface provided in HMD-VR could lead to better neurofeedback performance compared to the same feedback provided on a standard computer screen. In addition, differences in embodiment and presence between Screen and HMD-VR conditions were examined. Finally, we explored whether individual differences in embodiment and presence related to neurofeedback performance in each condition. 

Overall, we found preliminary evidence that healthy participants showed similar levels of neurofeedback performance in both Screen and HMD-VR conditions; however, we found a trend for better performance in the HMD-VR condition. Additionally, participants reported greater embodiment in the HMD-VR versus Screen condition and higher reported levels of embodiment related to better neurofeedback performance in the HMD-VR condition only. These preliminary results suggest that HMD-VR-based neurofeedback may rely on an individual’s sense of embodiment for successful use and improved performance. This is in line with our theoretical framework, which is further detailed in [29,39], in which we propose that greater embodiment of a virtual avatar should lead to larger changes in neural activity in the direction of the desired feedback. These results suggest that greater embodiment of HMD-VR neurofeedback essentially augments the weight of the neurofeedback, creating a more effective and responsive closed-loop neurofeedback system. This has important implications for a number of more recent HMD-VR neurofeedback systems [16,40,41,42,43]. However, future studies should explore these findings with a larger sample size over a longer period of time.

### 4.1. Similar Neurofeedback Performance and Time to Complete Successful Trials Between a Computer Screen and HMD-VR

Regardless of condition (Screen, HMD-VR), we found that on average, individuals were able to accurately modulate their brain activity to successfully control a virtual arm on over 80 percent of trials. These results suggest that neurofeedback based on motor imagery, using biologically relevant stimuli, can occur either on a computer screen or in immersive virtual reality using a head-mounted display. However, as seen in Figure 4a,b, there is a trend towards better performance and faster time to complete a successful trial in the HMD-VR condition compared to the Screen condition, which may not allow for significance because of our limited dataset (further discussed in Section 4.7). This trend towards greater sensorimotor desynchronization can also be observed in the individual subject data (Supplementary Figures S1 and S2), with more individuals showing more sensorimotor activity for the HMD-VR condition than the Screen condition. Additionally, there is a larger range of interindividual variability in both performance and average time to complete a successful trial in the HMD-VR condition, suggesting that some individuals may benefit from HMD-VR compared to others. This suggestion is further supported by the correlation between performance and embodiment, in which we show that individuals who had greater embodiment had better performance in HMD-VR only (further discussed in Section 4.5). An important future question to examine is whether neurofeedback performance in a clinical population (e.g., individuals with stroke) also shows no differences between HMD-VR and computer screen conditions.

### 4.2. Similar Power Spectral Density Between a Computer Screen and HMD-VR

Similarly, regardless of condition (Screen, HMD-VR), we found that on average, individuals had similar levels of sensorimotor activity, as measured by PSD between 8-24 Hz and when divided into alpha and beta frequency bands. This was expected as the sensorimotor desynchronization used to calculate PSD was also used to drive the virtual arm in the task. However, similar to the performance results, we see a trend for greater desynchronization in the alpha band for the HMD-VR condition (Figure 5b). While we do not see a trend for greater desynchronization in the beta band for the HMD-VR condition (Figure 5c), these results may indicate a neurofeedback-based effect for the different displays, suggesting that feedback type may be able to alter brain activity. We also showed a significant relationship between PSD and neurofeedback performance, where increased desynchronization corresponded to increased performance.

### 4.3. Similar Simulator Sickness Between a Computer Screen and HMD-VR

As expected, regardless of condition (Screen, HMD-VR), we found that on average, individuals had similar reported levels of simulator sickness. These results were expected given that our experimental design did not involve factors found to result in adverse simulator sickness (e.g., high accelerated movements, jumping movements, long usage time, etc.) [44] and suggest that HMD-VR neurofeedback does not cause additional adverse effects beyond using a computer screen in healthy individuals.

### 4.4. A higher Level of Embodiment in HMD-VR Compared to a Computer Screen

After performing the neurofeedback task in each condition (Screen, HMD-VR), participants reported having higher levels of embodiment in HMD-VR compared to the computer screen. This is in agreement with previous research showing that HMD-VR is effective for inducing embodiment [21,45]. However, while it has been intuitively suggested that viewing a virtual body in HMD-VR should induce greater embodiment than viewing the same virtual body on a computer screen, to our knowledge, there has been little empirical evidence to demonstrate this. Here, we address this gap by providing evidence that HMD-VR does seem to in fact increase embodiment compared to a computer screen during a neurofeedback task.

### 4.5. Greater Embodiment is Related to Better Neurofeedback Performance in HMD-VR

In line with our hypothesis, we show that greater embodiment was positively related to better neurofeedback performance in HMD-VR. This uniqueness to HMD-VR could possibly be explained by an increased range of embodiment levels in the HMD-VR condition compared to the Screen condition. These results are consistent with previous research where embodiment has been shown to lead to neurophysiological and behavioral changes based on the virtual body’s characteristics, such as overestimating object distances after being given an elongated virtual arm in HMD-VR [20]. These results are also constant with previous research showing embodiment of a humanoid arm compared to a robotic arm improves motor imagery skills [46]. While our findings do not support causality, they are important because they suggest that embodiment may have the potential to improve an individual’s neurofeedback performance, and HMD-VR may be able to increase the level of embodiment of an individual, beyond that of a normal computer screen. This suggests that if individuals were to encounter a ceiling effect while controlling neurofeedback on a computer screen, they might be able to show greater improvements, beyond this ceiling, if they show greater embodiment in HMD-VR.

### 4.6. Future Clinical Implications

We designed REINVENT as an EEG-based BCI with HMD-VR neurofeedback for individuals with severe motor impairments, such as stroke. However, before exploring the effectiveness of this device in a population with severe motor impairments, we first examined whether providing neurofeedback in HMD-VR improves performance compared to receiving the same neurofeedback on a computer screen in healthy adults. Our findings suggest that increased embodiment may improve individuals’ neurofeedback performance, which could potentially improve patients’ recovery. Furthermore, our results suggest that HMD-VR may facilitate an increased level of embodiment, beyond what might be seen with traditional screen-based BCIs. 

The findings in this pilot study are important considering that a patients’ perception of BCI control can have an effect on their own perceived capabilities [3]. Patients exhibiting a lack of embodiment may experience distress or a loss of control [3], which could then stifle recovery. Therefore, it is important to measure patients’ sense of embodiment in EEG-based BCI therapy to avoid feelings of estrangement. Future work might explore whether measures of embodiment, administered prior to HMD-VR neurofeedback training, could predict embodiment and neurofeedback performance. If so, these “pre-assessments” of embodiment potential could be used to predict and personalize EEG-based BCI therapy. As previous brain–computer interfaces have been shown to have a positive change on muscle and sensorimotor brain activity in post-stroke individuals, even when using screen-based environments [47], we anticipate that embodiment in HMD-VR may lead to even greater improvements. However, as these data are preliminary, more data are needed to explore this hypothesis.

### 4.7. Limitations

Our pilot study has several limitations. First was the limited sample size of 12 individuals and the limited number of trials collected per condition (i.e., 30 trials per condition). However, even with this limited sample, we were still able to extract the PSD, calculate relative PSD to baseline, and find a significant relationship between PSD and neurofeedback performance. However, this limited power may have resulted in the lack of significance seen between conditions in performance, PSD, and presence, where we see trends towards increased performance, greater sensorimotor desynchronization, and increased presence in the HMD-VR condition compared to the Screen condition, but these trends do not reach significance. Future research should explore this with greater power both in the number of participants and in the number of trials collected. Additionally, as our study was a within-subject experimental design, we were not able to examine differences in age and gender, which could influence EEG-based BCI performance. In addition to increasing the number of participants and trials collected, future studies should also consider a between-subjects experimental design.

A second limitation was the use of only eight channels of dry electrodes to collect sensorimotor activity and the broad frequency band used (8-24 Hz). Given that our system was initially designed to provide a low-cost rehabilitation intervention, we chose to drive the neurofeedback-based device with a limited number of dry electrodes as previous studies have found dry electrodes to be suitable for neurofeedback applications [15,48]. However, we recognize that the signal quality of these electrodes can be noisy, and even though we were able to successfully extract power spectral density, in future studies, we plan to use higher quality electrodes (e.g., active gel electrodes), which would also allow us to narrow the frequency band and personalize the feedback across individuals. In addition, although the low resolution from eight channels, primarily clustered around bilateral sensorimotor regions, facilitated a faster application of the EEG cap, it also limited our post-hoc analyses. Future research studies should utilize more channels for higher resolution. This would enable topographical analyses of whole brain activity during neurofeedback training as well as the ability to examine brain activity in non-motor regions as control regions.

A third limitation was the use of two separate questionnaires to examine presence and embodiment. Although presence and embodiment are often discussed separately, there are certainly overlapping features of the two constructs (e.g., greater embodiment often leads to greater presence). Future work may consider the use of standardized questionnaires that combine measures of presence and embodiment into a single questionnaire, such as the recently proposed questionnaire by Gonzalez-Franco and Peck, 2018 [49]. 

A fourth limitation is that here we studied only healthy individuals. This is notable as the effects observed may be smaller than those of a clinical population, who may have more room to improve. Specifically, the healthy individuals in our study showed, on average, 80% accuracy with the based BCI within a short time frame, which may reflect their intact sensorimotor control. However, individuals with severe motor impairments may start with lower scores and have greater room for improvement due to damage to these same networks. Future work may examine extended training with the HMD-VR environment to see if it is possible for individuals to improve beyond their current levels with greater time in the environment, as well as the effects of embodiment on EEG-based BCI performance in individuals with stroke, which may provide a greater range of abilities and thus greater potential effects with immersive virtual reality. Additionally, future work may examine whether EEG-based BCI training has an effect on real world movements and whether these effects are different between computer screen or HMD-VR environments. Future work should build upon these modest results and explore the effects of embodiment on HMD-VR neurofeedback performance with large samples and in clinical populations.

## 5. Conclusions

This preliminary work suggests that individuals have higher levels of embodiment when given immersive virtual reality-based neurofeedback compared to the neurofeedback displayed on a computer screen. Furthermore, this increased sense of embodiment in immersive virtual reality neurofeedback has the potential to improve neurofeedback performance in healthy individuals over their performance on a computer screen. HMD-VR may provide a unique medium for improving EEG-based BCI performance, especially in clinical settings related to motor recovery. Future work will explore ways to increase presence and embodiment in immersive virtual reality and examine these effects on motor rehabilitation in patients with severe motor impairment.

## Figures and Tables

**Table 1:** Individual questions on Embodiment Questionnaire.

Type	Question	Referenced	Scoring Scale
Self	To what extent did you feel that the virtual arm was your own arm?	Own Arm	Not at all/Very much (1…10)
Self	How much did the virtual arm’s actions correspond with your commands?	Arms Actions	Not at all/Very much (1…10)
Self	To what extent did you feel if something happened to the virtual arm it felt like it was happening to you?	Happening to Arm	Not at all/Very much (1…10)
Self	How much control did you feel you had over the virtual arm in this virtual environment?	Amount of Arm Control	No control/Full control (1…10)
Self	How much did you feel that your virtual arm resembled your own (real) arm in terms of shape, skin tone or other visual features?	Resembled Arm	Not at all/Very much (1…10)
Self	Did the virtual arm seem bigger, smaller or about the same as what you would expect from your everyday experience?	Size of Arm	Smaller/Larger (1…10)
Spatial	To what extent did you feel like you were really located in the virtual environment?	Location	None/Completely (1…10)
Spatial	To what extent did you feel surrounded by the virtual environment?	Surrounded	None/Completely (1…10)
Spatial	To what extent did you feel that the virtual environment seemed like the real world?	Real World	None/Completely (1…10)
Spatial	To what extent did you feel like you could reach out and touch the objects in the virtual environment?	Reach Out and Touch	None/Completely (1…10)

## Supplementary Materials

The following are available online at https://www.mdpi.com/1424-8220/20/4/1204/s1, Supplementary Table S1: Mean, SD, and skewness for all variables, Supplementary Figure S1: Individual participant EEG activity for C3, Supplementary Figure S2: Individual participant EEG activity for C4.
