# Supplementary material for: Embodiment Is Related to Better Performance on a Brain–Computer Interface in Immersive Virtual Reality: A Pilot Study
Source: Sensors (Basel). 2020 Feb 22;20(4):1204. doi: 10.3390/s20041204 (PMC7070491; doi:10.3390/s20041204)
Supplement: Supplementary file 1 [file sensors-20-01204-s001.pdf]

Table S1. Mean, SD, and skewness for all variables.

-	Screen Condition			HMD-VR Condition		
-	**Mean**	**SD**	**Skewness**	**Mean**	**SD**	**Skewness**
Neurofeedback performance	80.95%	9.1%	-0.27	83.33%	14.9%	-0.32
Time to complete successful trials	4.347 s	1.17 s	0.26	3.996 s	2.41 s	0.07
PSD band (8-24 Hz)	-4.69	2.96	-0.07	-4.32	3.41	-0.48
Alpha band (8-12 Hz)	-1.84	2.90	0.26	-2.89	3.04	-0.37
Beta band (13-24 Hz)	-5.88	3.08	-0.19	-4.92	3.63	-0.46
Nausea	1.59	8.94	0.36	2.39	5.93	-0.15
Oculomotor	9.48	12.15	0.96	9.45	9.76	-0.22
Disorientation	4.64	17.13	2.39	3.48	8.65	2.22
Realism	30.00	6.35	0.41	33.00	6.40	1.07
Possibility to Act	18.17	3.70	-0.57	19.92	4.19	0.21
Quality of Interface	12.83	3.07	0.16	13.42	2.97	-0.32
Possibility to Examine	13.17	2.59	0.17	14.92	2.27	0.45
Self-Evaluation of Performance	10.0	1.95	0.69	11.00	2.13	-0.18
Embodiment	4.68	1.27	1.01	5.4	1.71	0.64
Self Embodiment	5.39	1.17	-0.13	5.43	1.76	0.17
Spatial Embodiment	3.60	2.04	1.23	5.35	2.00	0.29

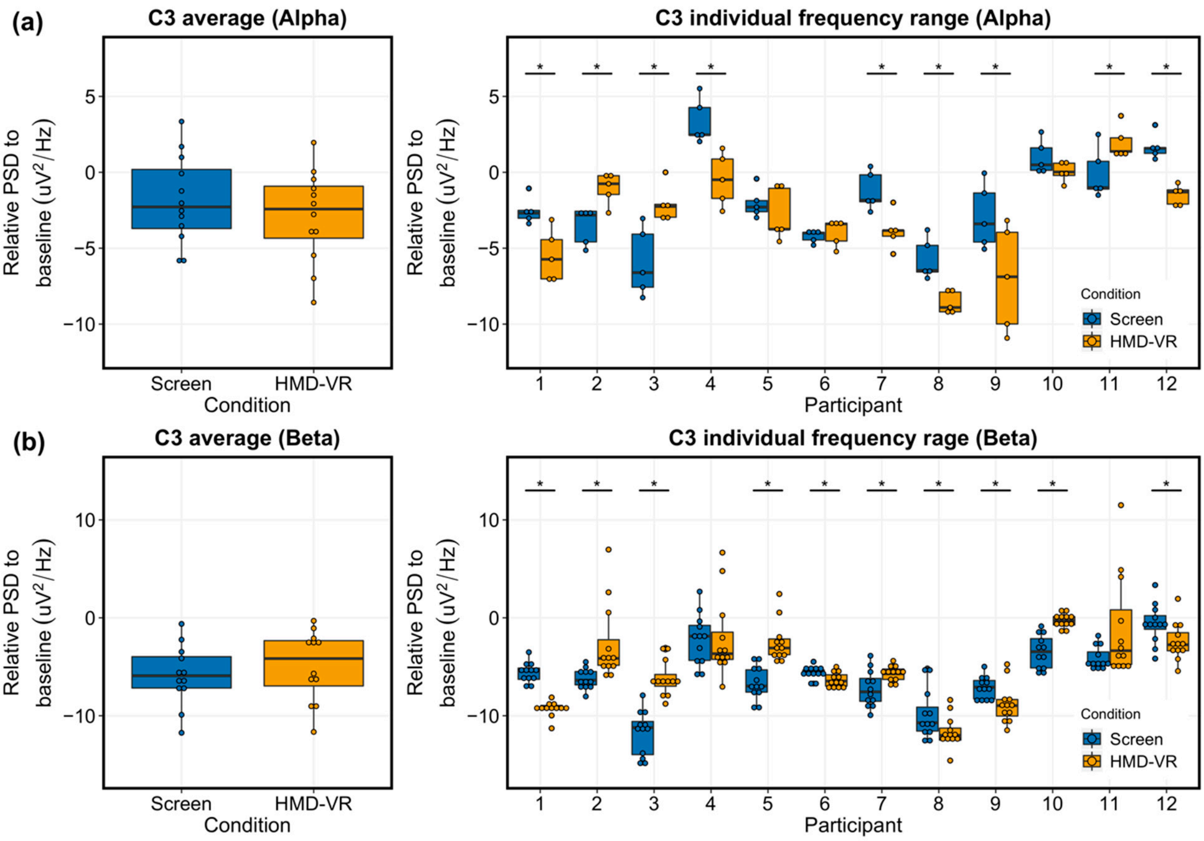

Figure S1. Individual participant EEG activity for C3. (a) Analysis of the relative PSD to baseline of C3 for the alpha band at the group-level (left) and at the individual-level (right). For individual subjects, for relative alpha levels during the task, there were significant differences between Screen and HMD-VR in nine participants. From those, three participants had significantly lower alpha (greater desynchronization) during the Screen condition and six participants had significantly lower alpha (greater desynchronization) during the HMD-VR condition. (b) Analysis of the relative PSD to baseline of C3 for the beta band at the group-level (left) and at the individual-level (right). For individual subjects, for relative beta levels during the task, there were significant differences between Screen and HMD-VR in ten participants. From those, five participants had significantly lower beta during the Screen condition and five participants had significantly lower beta during the HMD-VR condition.

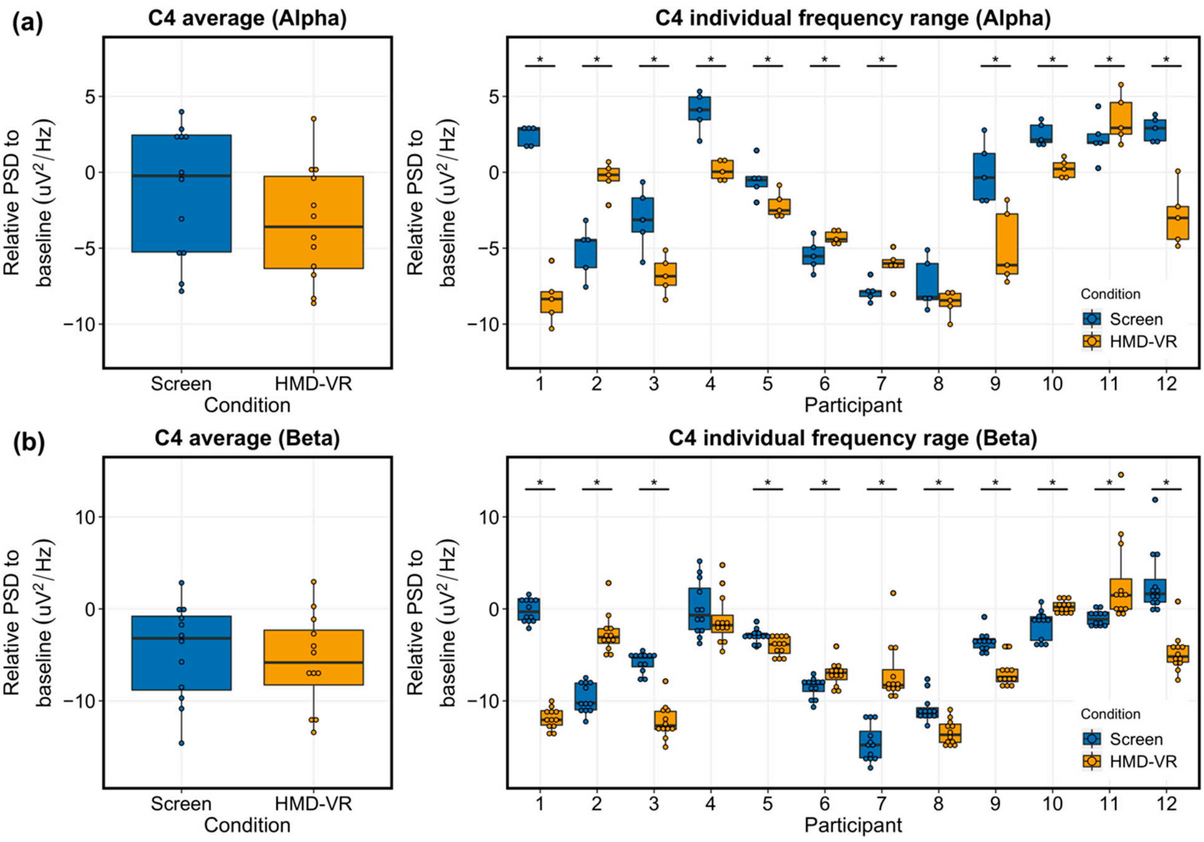

Figure S2. Individual participant EEG activity for C4. (a) Analysis of the relative PSD to baseline of C4 for the alpha band at the group-level (left) and at the individual-level (right). There were significant differences between Screen and HMD-VR in eleven participants. From those, four participants had significantly lower alpha during the Screen condition and seven participants had significantly lower alpha during the HMD-VR condition. (b) Analysis of the relative PSD to baseline of C4 for the beta band at the group-level (left) and at the individual-level (right). There were significant differences in the beta band between Screen and HMD-VR in eleven participants. From those, five participants had significantly lower beta during the Screen condition and six participants had significantly lower beta during the HMD-VR condition.
